# Neonatal unit admission and offspring mental health trajectories across childhood and adolescence: a nationally representative UK cohort study

**DOI:** 10.1136/bmjpo-2024-003092

**Published:** 2025-01-28

**Authors:** Madura Nandakumar, Gemma Lewis, Glyn Lewis, Francesca Solmi, Ramya Srinivasan

**Affiliations:** 1 Division of Psychiatry, University College London, London, UK

**Keywords:** Neonatology, Psychology, Child Psychiatry, Epidemiology

## Abstract

**Objective:**

To investigate the associations between neonatal unit admission (NNU) and subsequent emotional and behavioural difficulties during childhood and adolescence.

**Design:**

Longitudinal general population cohort study.

**Setting:**

The Millennium Cohort Study: nationally representative UK-based cohort.

**Participants:**

All children with exposure, outcome and confounding data.

**Exposure:**

NNU admission was identified at 9 months by asking parents whether their baby was ‘taken to special care or neonatal or intensive care unit after birth’.

**Main outcome measures:**

Emotional and behavioural problems were assessed using the Strengths and Difficulties Questionnaire when children were 3, 5, 7, 11, 14 and 17 years. We explored the association between NNU admission and trajectories of emotional and behavioural problems using multilevel models with growth curves for outcome data between 3–17 years and adjusted for a broad range of confounders.

**Results:**

14 013 participants (48.9% female, 13.7% ethnic minority) were included in the analytical sample. In the sample, mean gestational age was 275.81 (SD): 13.80) days, and mean birth weight was 3.36 kg (SD=0.58). 1273 (9.1%) participants had an NNU admission. The latter was associated with increased emotional difficulties (mean difference (MD) 0.13, 95% CI 0.045 to 0.22, p=0.003) and peer problems (MD 0.11, 95% CI 0.026 to 0.19, p=0.010) during childhood in fully adjusted models. There was no evidence that NNU admission was associated with conduct problems (MD 0.013, 95% CI −0.062 to 0.088, p=0.732) or hyperactivity symptoms (MD 0.042, 95% CI −0.070 to 0.15, p=0.452).

**Conclusions:**

Children admitted to NNUs at birth are more likely to experience emotional difficulties and peer problems during childhood. These differences are apparent from early childhood continuing into adolescence and strengthen the case for a calm NNU environment with parental visits and mental health support, and early interventions for children admitted to NNUs.

**WHAT IS ALREADY KNOWN ON THIS TOPIC**

- Neonatal units (NNUs) provide care for infants soon after they are born. One in seven infants in the UK is admitted to NNUs at birth and many of these infants are full-term and normal birth weight. Despite this, little is known regarding the mental health of children admitted to NNUs.

**WHAT THIS STUDY ADDS**

- In this analysis of a large national prospective birth cohort followed up to the end of adolescence, we found that children whose parents reported that they had been admitted to a special care or neonatal or intensive care unit after birth, were more likely to experience higher levels of emotional difficulties and peer problems during childhood and adolescence after. These findings suggest that the increased risk of emotional difficulties in those admitted to NNUs is not solely related to low birth weight, gestational age or pregnancy/delivery complications. There may be factors relating to NNU admission such as parent–infant separation, the NNU environment or parental stress/mental health which may be open to intervention, as well as supportive early interventions during early childhood to reduce these difficulties, adjusting for birth weight, gestational age and pregnancy/delivery complications.

**HOW THIS STUDY MIGHT AFFECT RESEARCH, PRACTICE OR POLICY**

- Although more research is needed to understand the mechanisms underlying the associations we observed, interventions to improve NNU environment, facilitation of parental visits and a focus on parental mental health support during and after admission may be of benefit.

## Introduction

 One in seven infants born in the UK requires admission to a neonatal unit (NNU) at birth^1^ including admission to neonatal intensive care (NICU) and special care baby units (SCBUs). Historically, most infants admitted to such units were preterm or low birth weight. In recent years, infants of normal birth weight and gestation have been increasingly admitted to NNUs,^2^ perhaps due to more admissions for low acuity diagnoses.^3^ In 2016, more than half of UK NNU admissions were infants born at term.^4^ Despite being a stressful experience for both the baby and the family,^5 6^ little is known about child mental health outcomes associated with NNU admission. Research has shown that premature and low birthweight children, often admitted to NNUs, might be at greater risk of neurodevelopmental problems,^7^ as well as emotional and behavioural problems in childhood.^8^,^11^ However, not all children admitted to NNU are preterm or low birth weight.

Only three cross-sectional studies have investigated mental health outcomes of children admitted to NNUs at birth with conflicting findings. A survey of 1140 Canadian NICU graduates compared with 393 full-term infants found that children admitted to NICU had worse behavioural, emotional and social functioning at 3.5 years of age.^12^ Similarly, a survey of 5520 Canadian children aged 4–17 years found that NICU graduates were more likely to experience psychiatric disorders during childhood and adolescence regardless of birth weight^13^ but did not account for prematurity. In contrast, a study of 160 Dutch NICU graduates aged 1–4 years, recruited from neonatology clinics, found no evidence of differences in problem behaviour or social functioning after adjusting for birth weight in those who had been admitted to NICU.^14^

These studies have limitations. They inconsistently accounted for important confounders including prematurity, birth weight, antenatal or perinatal complications and parental mental health, as well as sociodemographic factors including parental social class, parental education and ethnicity.^12^,^14^ Two studies were small which could result in low statistical power and type II error and selected participants based on prior NICU admission which might result in selection bias.^12 14^ Only one study measured mental health problems in adolescence^13^ which is the developmental period when they are most likely to emerge.^15^ However, its cross-sectional design did not allow a developmental investigation of when potential disparities in mental health problems may have arisen which is essential when considering timing of preventative interventions. Finally, only one study included infants admitted to SCBU,^13^ which comprise a significant proportion of NNU admissions; therefore, it is unclear if existing findings can be generalised to that population.

To address these limitations, we used data from a large representative UK cohort. We investigated differences in developmental trajectories of emotional and behavioural difficulties from age 3 to 17 years among participants who were admitted to an NNU at birth and those who were not adjusting for a broad range of confounders including birth weight, gestational age and pregnancy/delivery complications.

## Methods

### Population

We used data from the Millennium Cohort Study (MCS), an ongoing nationally representative, population-based birth cohort study of 18 818 children (from 18 551 families) born between September 2000 and January 2002 who were living in the UK at the age of 9 months (see online supplemental file 1). We included all children with complete data on exposure, confounders and at least one time point of outcome measurement. In multiple births, one child was included at random to account for the effect of shared genetic and environmental factors.

### Exposure

Infants admitted to NNUs at birth were identified at 9 months. Parents were asked whether the baby was ‘taken to special care or neonatal or intensive care unit after birth’. Maternal recall of such perinatal events has been found to be reliable.^16 17^

### Outcome

Emotional and behavioural problems in children were assessed using the Strengths and Difficulties Questionnaire (SDQ)^18^—a widely used and validated measure of emotional/behavioural difficulties in children^19 20^—at ages 3, 5, 7, 11, 14 and 17 years (see online supplemental file 1). We used the parent-reported SDQ as our primary outcome to ensure measurement comparability. As sensitivity analyses, we used three child-reported measures (the Short Moods and Feelings Questionnaire (sMFQ)^21^ at 14 years, the SDQ at 17 years and the Kessler psychological Distress Scale (Kessler-6)^22^ at age 17—see online supplemental file 1) to account for the fact that adolescents can reliably self-report and may have a different perspective to their parents.

### Confounders

We adjusted for several confounders measured at the first sweep of data collection which we selected based on their hypothesised association with the exposure and outcomes. Child-related factors were gestational age, birth weight, sex and ethnicity. Maternal/caregiver factors were parental social class, weekly income, maternal age at birth, maternal education and maternal lifetime history of depression. Lastly, we included the following pregnancy and labour factors: smoking in pregnancy, alcohol consumption in pregnancy, multiple pregnancy, whether antenatal care was given, prepregnancy BMI, complications in labour and pregnancy and delivery type (details in online supplemental file 1).

### Statistical analysis

Analyses were conducted in Stata V.16.^23^ We explored the association between NNU admission and trajectories of internalising and externalising symptoms at ages 3, 5, 7, 11, 14 and 17 years using multilevel models with time nested within individuals. Initially, we fit an unconditional model testing the association between linear and quadratic age variables and each outcome, centring age at the mean (model 1). We included NNU admission as the exposure variable in model 2. We progressively included confounders in multivariable models in a stepwise fashion. In final models, we included interaction terms between the exposure and time variables (models 7 and 8) to explore differences in trajectory slope by exposure. Detailed multilevel model specifications are described in online supplemental table 1. All analyses were conducted using weights accounting for the stratified nature of the sampling procedure. We ran a number of sensitivity analyses. First, we ran analyses using self-reported measures of depression (sMFQ) at age 14 years and self-reported SDQ scales at age 17 years. Second, we ran analyses using self-reported measures of depression (Kessler-10) at age 17 years. We included these analyses to investigate the potential for maternal reports of child mental health in adolescence to bias our analyses.

Third, we restricted analyses to participants born at 34 weeks of gestation to further exclude the potential for our results to be explained by mental health difficulties of extremely preterm babies. Lastly, we re-ran our models after imputing missing confounder data for participants. We did not impute outcome data, as the only children who would have been excluded from our models would have been those with no observed mental health data and as such we did not have any auxiliary variables that would have allowed us to robustly impute missing SDQ data. More details on these analyses and measures are provided in online supplemental file 1. The study has been reported according to Strengthening the Reporting of Observational Studies in Epidemiology guidelines for reporting of cohort studies (online supplemental material).

### Patient and public involvement

Public and participants were involved in the design of the MCS; patients and/or the public were not otherwise formally involved in the design or conduct or reporting or dissemination of this specific analysis. However, one of the study authors had experience of NICU admission during the conduct of this study.

## Results

### Sample

**Figure 1:**
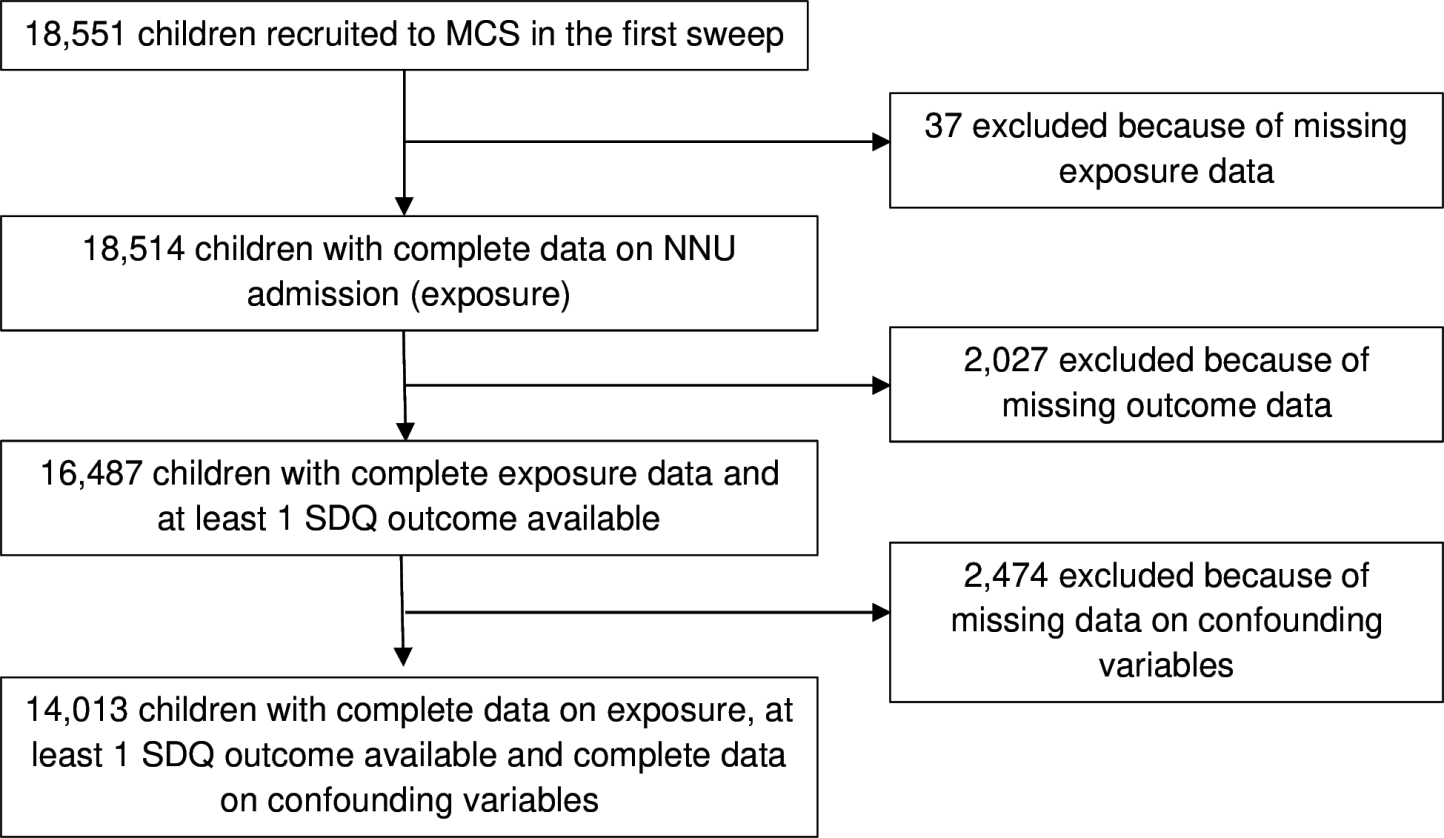
Study profile. MCS, Millennium Cohort Study; NNU, neonatal unit; SDQ, Strengths and Difficulties Questionnaire.

Of the 18 551 families recruited to the first sweep, exposure data were available for 18 514 (99.80%) children. Of these, 16 492 (89.07%) had at least one time point of outcome data available and 14 013 (75.54%) had data on all confounders and were included in the analytical sample (figure 1). Of those in the analytical sample, 11 780 (84.40%) had outcome data at 3 or more SDQ time points.

A greater proportion of children in the analytical sample were male and of white ethnicity. Mean gestational age was 275.81 days (SD: 13.80 days) and mean birth weight of 3.36 kg (SD: 0.58 kg).

A total of 1273 (9.08%) children were admitted to an NNU. Compared with children not admitted, a greater proportion of those admitted were male, white, had a mother with a history of depression, were part of a multiple pregnancy, born at a lower gestational age and birth weight and had encountered more complications in pregnancy/labour (table 1).

**Table 1:** Sociodemographic and clinical characteristics of participants included in the analytical sample, presented for the overall sample (n=14 013) and stratified by exposure status

Participants’ characteristic	Analytical sample	NNU admission	
		Not admitted	Admitted
	**N (%)**	**n (%)**	**n (%)**
Participants	14 013 (100)	12 740 (90.92)	1273 (9.08)
Sex:			
Female	6858 (48.94)	6323 (92.20)	535 (7.80)
Male	7155 (51.06)	6417 (89.69)	738 (10.31)
Ethnicity:			
White	12 099 (86.34)	10 986 (90.80)	1113 (9.20)
Black	346 (2.47)	318 (91.91)	28 (8.09)
South Asian	1001 (7.14)	912 (91.11)	89 (8.89)
Mixed	409 (2.92)	378 (92.42)	31 (7.58)
Other	158 (1.13)	146 (92.41)	12 (7.59)
Parental social class:			
Higher	9022 (64.38)	8212 (91.02)	810 (8.98)
Lower	4991 (35.62)	4528 (90.72)	463 (9.28)
Maternal education:			
Compulsory	8834 (63.04)	8036 (90.97)	798 (9.03)
Non-compulsory	5179 (36.96)	4704 (90.83)	475 (9.17)
Maternal history of depression:			
No	10 506 (74.97)	9632 (91.68)	874 (8.32)
Yes	3507 (25.03)	3108 (88.62)	399 (11.38)
Maternal smoking in pregnancy:			
Never smoked	9075 (64.76)	8275 (91.18)	800 (8.82)
Smoked but quit in pregnancy	1830 (13.06)	1651 (90.22)	179 (9.78)
Smoked in pregnancy	3108 (22.18)	2814 (90.54)	294 (9.46)
Maternal alcohol consumption during pregnancy:			
None	9730 (69.44)	8819 (90.64)	911 (9.34)
<=2 occasions per month	3033 (21.64)	2782 (91.72)	251 (8.28)
>2 occasions per month	1250 (8.92)	1139 (91.12)	111 (8.88)
Multiple pregnancy:			
No	13 944 (99.51)	12 697 (91.06)	1247 (8.94)
Yes	69 (0.49)	43 (62.32)	26 (37.68)
Antenatal care:			
Yes	13 621 (97.20)	12 397 (91.01)	1224 (8.99)
No	392 (2.80)	343 (87.50)	49 (12.50)
Type of delivery:			
Vaginal	9475 (67.62)	8908 (94.02)	567 (5.98)
Assisted	1438 (10.26)	1294 (89.99)	144 (10.01)
Planned caesarean	1327 (9.47)	1168 (88.02)	159 (11.98)
Emergency caesarean	1773 (12.65)	1370 (77.27)	403 (22.73)
	**Mean (SD**)	**Mean (SD**)	**Mean (SD**)
Gestational age (in days)	275.81 (13.80)	277.56 (10.52)	258.33 (25.55)
Birth weight (in kg)	3.36 (0.58)	3.41 (0.51)	2.87 (0.92)
Maternal age (years)	29.54 (5.77)	29.53 (5.76)	29.60 (5.93)
Weekly OECD equivalised income	310.19 (199.87)	310.44 (199.26)	307.67 (205.95)
Maternal prepregnancy BMI	23.74 (4.44)	23.70 (4.37)	24.15 (5.14)
Number of pregnancy complications	0.56 (0.88)	0.53 (0.84)	0.91 (1.11)
Number of labour complications	0.41 (0.69)	0.38 (0.67)	0.70 (0.83)

BMI, body mass index; NNU, neonatal unit; OECD, Organisation for Economic Co-operation and Development

A total of 4538 (24.46%) participants had missing data on either the exposure or confounders or did not have any outcome measurements available. The proportion of children admitted to NNUs did not differ between those included (9.08%) and excluded (9.06%) from analyses, but the latter were more likely to be males (52.34% vs 51.06%), from ethnic minority backgrounds (28.96% vs 13.66%), and to have mothers with only compulsory education (77.60% vs 63.04%), who had smoked in pregnancy (25.11% vs 22.18%) and had not attended antenatal care (6.92% vs 2.80%, table 2).

**Table 2:** Baseline characteristics of those included in the analytical sample (participants with complete exposure, confounders and at least one SDQ outcome available), and those with missing data

Characteristic	Included in analytical sample	
	Yes	No
	n (%)	n (%)
(% missing in those with exposure data)	14 013 (75.55)	4538 (24.46)
Neonatal unit admission:		
No/not applicable	12 740 (90.92)	4093 (90.94)
Yes	1273 (9.08)	408 (9.06)
Sex (0.00% missing data)		
Female	6858 (48.94)	2163 (47.66)
Male	7155 (51.06)	2375 (52.34)
Ethnicity (0.06% missing data)		
White	12 099 (86.34)	3216 (71.04)
Black	346 (2.47)	322 (7.11)
South Asian	1001 (7.14)	735 (16.23)
Mixed	409 (2.92)	145 (3.21)
Other	158 (1.13)	109 (2.41)
Parental social class (6.32% missing data)		
Higher	9022 (64.38)	1742 (51.77)
Lower	4991 (35.62)	1623 (48.23)
Maternal education (0.44% missing data)		
Compulsory	8834 (63.04)	3442 (77.60)
Non-compulsory	5179 (36.96)	994 (22.40)
Maternal history of depression (0.03% missing data)		
No	10 506 (74.97)	3468 (76.84)
Yes	3507 (25.03)	1045 (23.16)
Maternal smoking (0.15% missing data)		
Never smoked	9075 (64.76)	2847 (63.11)
Smoked but quit in pregnancy	1830 (13.06)	531 (11.77)
Smoked in pregnancy	3108 (22.18)	1133 (25.11)
Maternal alcohol consumption during pregnancy (0.18% missing data)		
None	9730 (69.44)	3442 (76.88)
<=2× per month	3033 (21.64)	693 (15.57)
>2× per month	1250 (8.92)	336 (7.5)
Multiple pregnancy (0.01% missing data)		
No	13 944 (99.51)	4485 (99.56)
Yes	69 (0.49)	20 (0.44)
Antenatal care (0.21% missing data)	Antenatal care:	
Yes	13 621 (97.20)	4157 (93.08)
No	392 (2.80)	309 (6.92)
Type of delivery (0.32% missing data)	Type of delivery:	
Vaginal	9475 (67.62)	3107 (76.06)
Assisted	1438 (10.26)	385 (9.42)
Planned caesarean	1327 (9.47)	406 (9.93)
Emergency caesarean	1773 (12.65)	547 (13.39)
	**Mean (SD)**	**Mean (SD)**
Gestational age (days) (1.24% missing data)	275.81 (13.80)	275.43 (14.85)
Birth weight (kg) (0.23% missing data)	3.36 (0.58)	3.29 (0.61)
Maternal age (years) (0.21% missing data)	29.54 (5.77)	27.86 (6.33)
Weekly OECD equivalised income (1.0% missing data)	310.23 (199.87)	217.88 (164.66)
Pre-pregnancy BMI (8.81% missing data)	23.74 (4.44)	23.21 (4.60)
Number of pregnancy complications (0.04% missing data)	0.56 (0.88)	0.48 (0.80)
Number of labour complications (0.04% missing data)	0.41 (0.69)	0.34 (0.62)

BMI, body mass index; OECDOrganisation for Economic Co-operation and DevelopmentSDQStrengths and Difficulties Questionnaire

### Internalising problems

Results of unconditional models are presented in online supplemental table 2.

**Figure 2:**
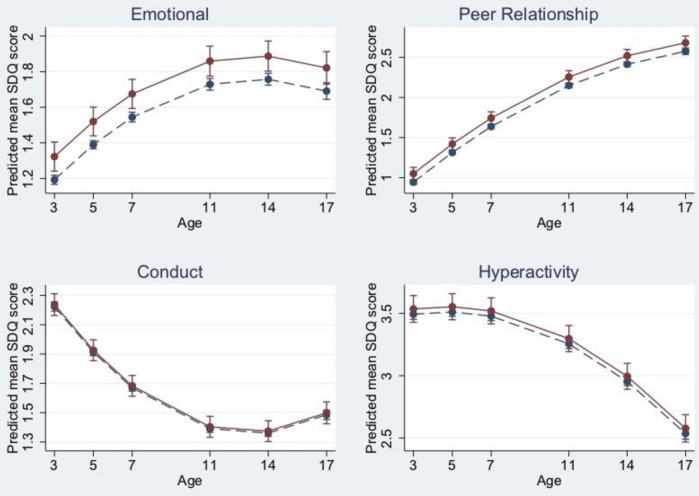
Trajectories of SDQ subscale scores for children admitted (solid line) and not admitted (dashed line) to NNU with 95% CIs. Trajectories derived from the fully adjusted model. NNU, neonatal unit; SDQ, Strengths and Difficulties Questionnaire.

In unadjusted models, children admitted to NNU had on average greater emotional symptoms (model 2 mean difference (MD) 0.23, 95% CI 0.15 to 0.31, p<0.001) and peer problems (model 2 MD 0.22, 95% CI 0.14 to 0.30, p<0.001) compared with those not admitted. Although the magnitude of these associations was attenuated after inclusion of all confounders, there was still strong evidence of an association with emotional symptoms and peer problems (emotional symptoms MD 0.13, 95% CI 0.05 to 0.22, p=0.003; peer problems MD 0.11, 95% CI 0.03 to 0.19, p=0.010) (see table 3). We did not find any evidence of interaction between NNU admission and linear or quadratic time variables for emotional problems (p=0.606 and p=0.538, respectively), and peer problems (p=0.271 and p=0.126, respectively) (figure 2).

**Table 3:** Results of unadjusted and adjusted multilevel models showing the mean differences in parent-reported SDQ scores according to NNU admission in a sample with complete exposure, outcome and confounding data (n=14 016) using population weights

Regression models:	Outcome: child mental health problems aged 3–17 years, measured with SDQ subscales			
	Emotional symptoms	Peer problems	Conduct problems	Hyperactivity
	Mean difference(95% CI), p value	Mean difference(95% CI), p value	Mean difference(95% CI), p value	Mean difference(95% CI), p value
Model 2: NNU admission+time variables mean centred age and age squared*	0.23 (0.15 to 0.31), <0.0001	0.22 (0.14 to 0.30),<0.0001	0.08 (0.01 to 0.15),0.023	0.25 (0.15 to 0.36),<0.0001
Model 3: Model 2+gestational age, birth weight, sex and ethnicity	0.18 (0.09 to 0.26),<0.0001	0.13 (0.05 to 021),0.002	−0.002 (−0.08 to 0.08),0.946	0.05 (−0.06 to 0.16),0.366
Model 4: Model 3+parental social class, weekly income, maternal age at birth and maternal education	0.19 (0.11 to 0.28),<0.0001	0.15 (0.07 to 0.23),<0.0001	0.02 (−0.05 to 0.10),0.590	0.08 (−0.02 to 0.19),0.127
Model 5: Model 4+maternal lifetime history of depression, smoking in pregnancy, alcohol consumption in pregnancy, multiple pregnancy, whether antenatal care was given, prepregnancy BMI	0.17 (0.09 to 0.26), <0.0001	0.14 (0.06 to 0.22),0.001	0.01 (−0.06 to 0.08),0.785	0.07 (−0.04 to 0.18),0.213
Model 6: Model 5+complications in labour and pregnancy and delivery type	0.13 (0.04 to 0.22), 0.003	0.11 (0.03 to 0.19), 0.010	0.01 (−0.06 to 0.09), 0.732	0.04 (−0.07 to 0.15), 0.452

* Time variables were: mean centred age and age squared. These were included in an unconditional model (Mmodel 1, shown in Supplemental Table 2online supplemental table 2) to investigate the trajectory of each mental health difficulties’ domain across childhood and adolescence.

BMIbody mass indexNNUneonatal unitSDQStrengths and Difficulties Questionnaire

### Externalising problems

In unadjusted models, children who had been admitted to NNU had greater conduct problems (MD 0.084, 95% CI 0.012 to 0.16, p=0.023) and hyperactivity problems (MD 0.25, 95% CI 0.14 to 0.35, p<0.0001). However, after inclusion of all confounders, there was no longer evidence of an association between NNU admission and conduct problems or hyperactivity (conduct problems MD 0.013, 95% CI −0.062 to 0.088, p=0.732; MD hyperactivity 0.042, 95% CI −0.070 to 0.15, p=0.452) (see table 3). There was weak evidence of an interaction between NNU admission and linear age for conduct problems (p=0.114, online supplemental figure 1) and no evidence for hyperactivity (p=0.917). There was no evidence for quadratic age with both outcomes (conduct p=0.629, hyperactivity p=0.563, figure 2).

### Sensitivity analyses

In sensitivity analyses (imputed sample, n=14 013), investigating the association between NNU admission and self-reported depressive symptoms at age 14 years using the sMFQ, there was evidence that children admitted to NNU had higher depressive symptoms at 14 years in fully adjusted models (MD 0.56, 95% CI 0.04 to 1.08, p=0.034, online supplemental table 3). At age 17 years, we did not find any evidence of an association between NNU admission and self-reported SDQ scores on the emotional difficulties, peer problems, hyperactivity or conduct problems subscales in unadjusted or adjusted models (online supplemental table 4). Children admitted to NNU had higher conduct problems: (MD 0.16, 95% CI −0.08 to 0.38, p=0.181); however, there was no statistical evidence for this association, in line with the findings from the main analyses. Similarly, in line with results of main analyses, we observed that children admitted to NNU had greater depressive symptoms as measured using the Kessler-6 at age 17 year compared with those not admitted; however, there was no statistical evidence for this association (online supplemental table 3). When we restricted analyses to participants born at 34+ weeks of gestation (n=13 746) and when we re-ran our models in a sample of participants with compete exposure and at least one outcome measure available, but imputed confounder data (n=16 487), the results were unchanged from those presented in the main analyses (online supplemental tables 4 and 5).

See online supplemental file 1 for full results of sensitivity analyses.

## Discussion

This is the first study to prospectively examine the association between NNU admission and subsequent trajectories of emotional and behavioural difficulties through childhood and adolescence. We found evidence that those admitted to NNUs were more likely to have consistently higher emotional difficulties and peer problems (internalising symptoms) throughout childhood based on parental reports. We found no evidence that these children had more conduct problems or higher hyperactivity scores (externalising symptoms). This is consistent with the hypothesis that NNU admission is a risk factor for the development of subsequent emotional difficulties independent of birth weight, gestational age and pregnancy/delivery complications.

The differences in internalising symptom scores between those admitted to NNU and the unexposed group were consistent between the ages of 3 and 17 years, with minimal divergence in trajectories. This suggests that these problems emerge early in childhood in this population, and early intervention could perhaps help to reduce the disparity between groups throughout childhood. Our main findings were supported by our sensitivity analyses at age 14 years where we found evidence of greater depressive symptoms at age 14 years using the child-reported sMFQ. We found evidence of an association between NNU admission and emotional problems at 17 years based on parental report but did not find clear evidence of an association based on either the self-reported Kessler-6 scale or the self-reported SDQ at 17. However, these sensitivity analyses have lower power than the multilevel models used in the main analyses, and the CIs included the values of the results from the multilevel models, so we cannot definitively conclude that these two sets of estimates are different. In addition, we found no evidence of interactions between NNU admission and the time variables in the multilevel models, indicating that the associations identified did not differ according to participants’ age. Based on our results, it is likely that there are ongoing differences at 17 years which our sensitivity analyses, were not sufficiently powered to identify but the possibility that the effects of NNU admission diminish by the age of 17 also needs to be considered. This requires further investigation in future studies.

### Limitations

We limited our analytical sample to participants with complete data on exposure, confounders and at least one time point of outcome data available. While this might introduce potential for selection bias, our sample still included the majority of MCS participants (ie, >75%) and there were no differences in NNU admissions between participants included and excluded from the analyses. To further reduce potential for bias, we also adjusted our models for variables associated with missing outcome data.

Although we controlled for several observed confounders, we cannot exclude the potential for unobserved confounding, including family history of mental health problems, to bias our results. Although we included maternal lifetime history of depression, we were not able to adjust for other maternal mental health problems such as anxiety and stress which may influence pregnancy and future offspring mental health.^24^,^26^ We were also not able to exclude genetic confounding, so future studies should use genetically informed designs. Similarly, we were unable to directly control for the reason the child required NNU admission, which could include brain injuries or genetic conditions, particularly in children born at term and are also likely to be higher in those admitted to NNU. While we have included a number of variables which might be associated with these outcomes (eg, maternal age, antenatal visit attendances and pregnancy and birth complications) we cannot exclude the presence of brain injuries or genetic conditions as a potential confounder of these associations. Furthermore, if these infants are more likely to experience repeated hospitalisations and if those were associated with the outcomes under investigation as opposed to NNU in itself our results might be affected by additional residual confounding.

In our main analyses, we used parent-reported SDQ as the outcome measure. These are more appropriate when children are younger, but as children become older and enter adolescence self-reported measures are preferable. In the MCS, self-reported SDQ scores are available at age 17 but their inclusion could have complicated result interpretation due to the change in reporter. To address this potential limitation, we conducted sensitivity analyses using data from available self-reported measures of depressive symptoms at 14 and 17 years, and of the self-reported SDQ at age 17 years. We were reassured to see that our findings in relation to depressive symptoms were in line with those of parental-reported internalising symptoms in the main analyses, although our models might have been underpowered to detect differences at age 17 years.

Our sample was a nationally representative UK-based cohort, so while it is representative of the UK population, the results may not be generalisable to other countries or healthcare systems; further research across international settings would be beneficial to allow cross-cultural comparisons.

### Comparison with previous studies and possible mechanisms

Our findings are in line with most,^12 13^ but not all,^14^ previous studies investigating child and adolescent mental health outcomes of NNU populations; we found an association with internalising symptoms despite including a broader population (NICU and SCBU) and adjusting for a broader set of confounders, including birthweight gestational age and pregnancy/delivery complications. In contrast with some studies,^13^ we did not find an association between NNU admission and externalising symptoms; this may be because we accounted for factors which are associated with neurodevelopmental differences such as low birth weight, gestational age and pregnancy complications.^7^,^11^ Infants are highly dependent on their caregivers for survival and parents provide infants with physiological and emotional regulation via close physical contact. The period of early separation and lack of close physical contact between parents and child may be a potential mediator of the association we observe^27^ as this has been associated with problems with emotional development and in developing relationships.^28 29^ Infants may also be subject to painful or intrusive procedures which may be distressing to them, particularly without the close contact of a caregiver to assist with both physiological and emotional regulation.^28^ In NNUs, children are exposed to repeated noise from equipment alarms, closing of incubator windows, blood bottles placed on incubators and nearby conversations.^30^ Exposure to ambient noise levels has been found to have several detrimental effects to infants in NICU, including sleep disturbance, apnoeas and physiological changes in response to stress such as changes to heart rate, respiratory rate, oxygen saturation and blood pressure.^31 32^ Similarly, there could be increased infant stress from continuous, bright, non-Circadian light levels experienced in NNUs.^33^ Finally, having a child admitted to an NNU is also a stressful experience for parents^5 6^; this may affect their mental health with possible negative effects on their child’s mental health.^34^ Further qualitative work to understand parent and child perspectives of how NNU may affect longer-term mental health and to guide further research and to develop interventions to mitigate the impact of NNU admission would be beneficial.

Children admitted to NNU are at risk of subsequent internalising problems and these differences seem to emerge early on in life and remain consistent through childhood and adolescence. If the associations we observed are causal, our findings suggest that this group of children and their parents could benefit from early interventions to prevent emotional problems. NNU admission is a complex, multimodal, life-saving intervention; however, if our results are causal, they suggest that there may be factors associated with the NICU admission that may have a long-lasting impact on children’s risk of internalising symptoms. Given that admission to NICU is not optional, our findings suggest that modifying the intervention to ensure it is less disruptive to neonates and their families and offering supportive early interventions, may help to prevent emotional problems. Further work on developing such interventions could include adjusting NICU and SCBU light and noise levels and facilitating parental visiting with a focus on mental healthcare for parents.

## supplementary material

10.1136/bmjpo-2024-003092 online supplemental file 1

## Data Availability

Data are available in a public, open access repository.
