# Supplementary material for: Neonatal unit admission and offspring mental health trajectories across childhood and adolescence: a nationally representative UK cohort study
Source: BMJ Paediatr Open. 2025 Jan 28;9(1):e003092. doi: 10.1136/bmjpo-2024-003092 (PMC11781137; doi:10.1136/bmjpo-2024-003092)
Supplement: online supplemental file 1 [file bmjpo-9-1-s001.pdf]

Neonatal unit admission and child mental health trajectories in a nationally representative UK cohort

Madura Nandakumar[1], Gemma Lewis[2], Glyn Lewis[2], Francesca Solmi*[2], Ramya Srinivasan*[2]

[1] UCL Division of Psychiatry, London, UK

** These authors contributed equally to this work*

Corresponding author:

Dr Ramya Srinivasan

UCL Division of Psychiatry,
Maple House, 6th floor, wing A
149 Tottenham Court Road
W1T 7NF, London, UK

Email: ramya.srinivasan.12@ucl.ac.uk

Supplemental Materials

Table of Contents

Study population, design and setting ... 3

Outcome measure: the Strengths and Difficulties Questionnaire (SDQ) ... 3

Outcome measures used in sensitivity analyses: the sMFQ and Kessler-6 psychological distress scale ... 4

Confounders ... 4

Statistical Analyses ... 6

Supplementary table 1: Specification for multilevel models ... 7

Statistical analyses: sensitivity analyses and missing data ... 8

STROBE Statement—Checklist of items that should be included in reports of *cohort studies* ... 9

Results ... 11

Supplementary table 2: M1 ... 13

Supplemental Figure 1 ... 14

Results from sensitivity analyses based on child-reported measures in adolescence ... 15

Child-reported depressive symptoms at 14 years as measured by the sMFQ ... 15

Child-reported psychological distress at 17 years as measured by the K6 ... 15

Supplemental table 3: MFQ and Kessler 6 ... 16

Supplemental Table 4: Analyses restricted to children born after 34 weeks of gestation ... 17

Supplemental Table 4: Analyses in multiply imputed dataset ... 17

Child-reported emotional and behavioural difficulties at 17 years as measured by the SDQ ... 18

Supplementary table 4: Child-reported SDQ ... 19

References ... 20

Study population, design and setting

The MCS over-sampled children from disadvantaged and ethnic minority groups, allowing them to be adequately represented. There have been seven main sweeps of data collection to date, initially at nine months of age, then at three, five, seven, 11, 14 and 17 years of age. Caregivers were surveyed at each sweep using self-completed questionnaires and face to face interviews, excluding the final sweep at age 17 where caregivers completed an online questionnaire only. Participants themselves were also included in interviews from age 7 onwards. Ethical approval for each wave of the study was granted by the National Health Service Multi Research Ethics Committee. Written, informed consent was provided by parents and, from wave four (i.e. age seven years) onwards, consent was also obtained from children. The full details of the study, recruitment and follow-up can be found on the MCS website (https://cls.ucl.ac.uk/cls-studies/millennium-cohort-study/).

Outcome measure: the Strengths and Difficulties Questionnaire (SDQ)

The SDQ is a 20-item screening questionnaire covering emotional difficulties and peer problems (internalising problems), and conduct problems and hyperactivity (externalising problems). Each of these four subscales is measured with five questions scored on a Likert scale scored 0 (not true), 1 (somewhat true), and 2 (certainly true), giving a total score ranging from 0 to 10. Higher scores indicate greater difficulties. From ages three to 14, the SDQ questionnaire was parent-completed; at the age of 17, the questionnaire was completed by both the parent and the adolescent.

Outcome measures used in sensitivity analyses: the sMFQ and Kessler-6 psychological distress scale

The sMFQ (1) is a 13-item screening tool for depression in young people, measuring depressive symptom severity in the preceding two weeks as outlined in the diagnostic and statistical manual of mental disorders (DSM-IV), which has been shown to be a valid measure to detect depression in this age group (2,3). The 6-item K6 (4) scale quantifies symptoms relating to the preceding 30 days and has been found to be a robust measure of internalising symptoms in adolescence (5-9)

Confounders

Child-related factors included were: gestational age (in days) derived from linked hospital records, birthweight (in kilograms) as reported by the caregiver, sex (male/female) and ethnicity (white, Black African or Caribbean, South Asian, mixed, and other including Chinese and other Asian) according to UK census definitions. Birthweight and gestational age in the sample were moderately correlated (0.59). As a result, given these variables are being considered only as confounders, to effectively reduce confounding, we included both variables in the models. This is because moderate collinearity between two confounding variables does not typically interfere with the estimation of the primary association of interest, between the exposure and outcome

Maternal/caregiver factors included were: parental social class (higher: managerial, intermediate occupations and self-employed, lower: technical, semi-routine and routine occupations) measured using the National Statistics Socio-economic Classification (10), weekly income (calculated using Organisation for Economic Co-operation and Development equivalence scales), maternal age at birth of the study child, maternal education (compulsory/non-compulsory) and maternal lifetime history of depression (no/yes). Lastly, we included pregnancy and labour factors: smoking in pregnancy (no, smoked but quit in pregnancy and smoked in pregnancy), alcohol consumption in pregnancy (never, less than twice per month, or more than twice per month), multiple pregnancy, whether antenatal care was given, pre-pregnancy BMI, complications in labour and pregnancy and delivery

type. The latter was coded as 'normal' (including water births), 'assisted' (including forceps and Ventouse), 'planned Caesarean section' and 'emergency Caesarean section'.

To account for complications during labour and pregnancy, we used a count of the total number of complications selected by respondents. Where there was overlap between certain pregnancy/labour complications and delivery type, the complication was only included in the count for one of the variables. For example, placenta praevia and raised blood pressure were listed as both pregnancy and labour complications, and so were only included in the former. Additionally, breech birth was not included as a labour complication if the mother went on to have a planned C-section. Pregnancy complications included: bleeding in later pregnancy, persistent severe vomiting, raised blood pressure/pre-eclampsia, urinary infection, diabetes/gestational diabetes, too much fluid around the baby, slow growth of baby, anaemia, blood group incompatibilities, other blood disorder, sciatica, symphysis pubis dysfunction, non-trivial infection, liver/gallbladder problems, asthma/eczema, depression/mental illness, neurological problems/epilepsy, threatened labour, early rupture of membranes, foetal heart slow/faint/inaudible, other suspected foetal problem, bleeding due to low lying placenta, to little fluid around the baby, accident or injury, other suspected problem. Labour complications included: footling breech presentation, other abnormal lie, very long labour, very rapid labour, foetal distress due to heart rate sign, foetal distress due to meconium, raised blood pressure, bleeding due to low lying placenta, bleeding due to abruption, other haemorrhage, cord around neck, occipitoposterior presentation, cephalopelvic disproportion, uterine inertia, delay in labour, other breech presentation, instrumental delivery, infection in labour, manual manipulation, early rupture of membranes.

Statistical Analyses

Initially, we fit an unconditional model testing the association between linear and quadratic age variables and each outcome, centring age at the mean to investigate overall symptom trajectories (model 1). Quadratic time variables were included in the final models if there was evidence of an association with the outcome. We specified random effects for intercept and slope, and an unstructured covariance matrix. We subsequently included neonatal unit admission as the exposure variable in model 2. Following this, confounding factors were progressively included in multivariable models. Initially, child related factors were added (model 3), followed by socio-economic and maternal demographics (model 4), prenatal factors (model 5) and perinatal factors (model 6). In the final models, we also included interaction terms between the exposure and time variables (models 7 and 8) to explore whether the association between exposure and outcome differed according to timepoint. Specifications for the multilevel models are summarized in supplementary table 1.

Supplementary table 1: Specification for multilevel models

Model	Specification
M1	Random slopes model (with unstructured covariance matrix) testing for association between time and outcome*, with time centred at the mean, including both linear and quadratic time variables – see supplementary table 2
M2	M1+ NICU admission as an explanatory variable (unadjusted model)
M3	M2 + child related factors: gestational age, birthweight, sex and ethnicity
M4	M3 + socio-economic and maternal demographics: parental social class, weekly income, maternal age at birth and maternal education
M5	M4 + maternal prenatal factors: maternal lifetime history of depression, smoking in pregnancy, alcohol consumption in pregnancy, multiple pregnancy, whether antenatal care was given, pre-pregnancy BMI
M6	M5 + maternal perinatal factors: complications in labour and pregnancy and delivery type
M7	M6 + interaction term between exposure and linear time variable
M8	M7 + interaction between exposure and quadratic time variable

* SDQ subscale scores at each timepoint, clustered within individuals

Statistical analyses: sensitivity analyses and missing data

Sensitivity analyses testing the association between neonatal admission and child reported internalising and externalising symptoms at 17 years, the SMFQ score at 14 years, and the Kessler-6 at 17 years, we used univariable and multivariable linear regression adjusting for the same set of confounders as the main analyses. Compared to those in the main analyses, these models had larger proportions of missing data and different complete case sample sizes, as they relied on only one time point of outcome data in adolescence.

We used multiple imputation to impute missing outcome data for children with complete exposure and confounding variable data and with at least one observed SDQ measurement (i.e., those included in the main analyses) to ensure that the analytical sample for these analyses was comparable to that of the main analyses.

STROBE Statement—Checklist of items that should be included in reports of *cohort studies*

	Item No	Recommendation	Page No
**Title and abstract**	1	(*a*) Indicate the study's design with a commonly used term in the title or the abstract	1
		(*b*) Provide in the abstract an informative and balanced summary of what was done and what was found	2
**Introduction**			
Background/rationale	2	Explain the scientific background and rationale for the investigation being reported	4-5
Objectives	3	State specific objectives, including any prespecified hypotheses	5
**Methods**			
Study design	4	Present key elements of study design early in the paper	1-2
Setting	5	Describe the setting, locations, and relevant dates, including periods of recruitment, exposure, follow-up, and data collection	5-6 & supplement
Participants	6	(*a*) Give the eligibility criteria, and the sources and methods of selection of participants. Describe methods of follow-up<br>(*b*) For matched studies, give matching criteria and number of exposed and unexposed	5-6 & supplement
Variables	7	Clearly define all outcomes, exposures, predictors, potential confounders, and effect modifiers. Give diagnostic criteria, if applicable	6-7 & supplement
Data sources/ measurement	8*	For each variable of interest, give sources of data and details of methods of assessment (measurement). Describe comparability of assessment methods if there is more than one group	5-7 & supplement
Bias	9	Describe any efforts to address potential sources of bias	6-7 & supplement
Study size	10	Explain how the study size was arrived at	5-6, fig1
Quantitative variables	11	Explain how quantitative variables were handled in the analyses. If applicable, describe which groupings were chosen and why	6-7 & supplement
Statistical methods	12	(*a*) Describe all statistical methods, including those used to control for confounding<br>(*b*) Describe any methods used to examine subgroups and interactions<br>(*c*) Explain how missing data were addressed<br>(*d*) If applicable, explain how loss to follow-up was addressed<br>(*e*) Describe any sensitivity analyses	7 & supplement

**Results**			
Participants	13*	(a) Report numbers of individuals at each stage of study—eg numbers potentially eligible, examined for eligibility, confirmed eligible, included in the study, completing follow-up, and analysed<br>(b) Give reasons for non-participation at each stage<br>(c) Consider use of a flow diagram	8, fig 1 and tables 1 & 2
Descriptive data	14*	(a) Give characteristics of study participants (eg demographic, clinical, social) and information on exposures and potential confounders<br>(b) Indicate number of participants with missing data for each variable of interest<br>(c) Summarise follow-up time (eg, average and total amount)	8 and tables 1 & 2
Outcome data	15*	Report numbers of outcome events or summary measures over time	Tables 1 & 2 and supplement
Main results	16	(*a*) Give unadjusted estimates and, if applicable, confounder-adjusted estimates and their precision (eg, 95% confidence interval). Make clear which confounders were adjusted for and why they were included<br>(*b*) Report category boundaries when continuous variables were categorized<br>(*c*) If relevant, consider translating estimates of relative risk into absolute risk for a meaningful time period	8-9 and table 3
Other analyses	17	Report other analyses done—eg analyses of subgroups and interactions, and sensitivity analyses	9-10 and supplement
**Discussion**			
Key results	18	Summarise key results with reference to study objectives	11
Limitations	19	Discuss limitations of the study, taking into account sources of potential bias or imprecision. Discuss both direction and magnitude of any potential bias	12-13
Interpretation	20	Give a cautious overall interpretation of results considering objectives, limitations, multiplicity of analyses, results from similar studies, and other relevant evidence	10-12
Generalisability	21	Discuss the generalisability (external validity) of the study results	12-13
**Other information**			
Funding	22	Give the source of funding and the role of the funders for the present study and, if applicable, for the original study on which the present article is based	15-16

*Give information separately for exposed and unexposed groups.

Note: An Explanation and Elaboration article discusses each checklist item and gives methodological background and published examples of transparent reporting. The STROBE checklist is best used in conjunction with this article (freely available on the Web sites of PLoS Medicine at http://www.plosmedicine.org/, Annals of Internal Medicine at http://www.annals.org/, and Epidemiology at http://www.epidem.com/). Information on the STROBE Initiative is available at http://www.strobe-statement.org.

Results

Internalising symptoms

Emotional difficulties increased between the ages of 3 and 14 for all children (model 1 estimated coefficient for a one unit increase in mean centred age: 0·042, 95% confidence interval [CI]: 0·038 to 0·045) and decreased at age 17 years (estimated coefficient for a one unit increase quadratic age: -0·0052, 95% CI: -0·0060 to -0·0045). On the other hand, peer problems increased between time points throughout the study period (model 1 estimated coefficient for a one unit increase in mean centred age: 0·12, 95% CI: 0·12 to 0·13), though at a lower rate in adolescence (estimated coefficient for a one unit increase in quadratic age: -0·0057, 95% CI: -0·0064 to -0·0050).

The magnitude of these associations was attenuated after inclusion of all covariates but there was still evidence of an association with both outcomes after adjustment for child characteristics in model 3 (MD emotional problems: 0·18, 95%CI: 0·09 to 0·26, p<0·0001; MD peer problems: 0·13, 95%CI: 0·05 to0.21, p=0·002). Despite there was still evidence of an association with both outcomes, its magnitude was further attenuated by inclusion of pregnancy-related complications for emotional symptoms (model 6 MD: 0·13, 95% CI: 0·05 to 0·22, p=0.003), whereas it remained largely unchanged for peer problems (model 6 MD: 0·11, 95% CI: 0·03 to 0·19, p=0.010). See Table 3.

Externalising symptoms

Conduct problems decreased between the ages of three and 11 years (model 1 estimated coefficient for a one unit increase in mean centred age: -0·064, 95% CI: -0·066 to -0·061),

and slightly increased again between the ages of 14 and 17 years (model 1 estimated coefficient for a one unit increase in quadratic age: 0·009, 95% CI: 0·008 to 0·009). On the contrary, hyperactivity scores remained stable until seven years of age and decreased between the ages of 7 and 17 (model 1 estimated coefficient for a one unit increase in mean centred age: -0·065, 95% CI: -0·068 to -0·061; quadratic time term MD: -0·0066, 95% CI: -0·0075 to -0·0057).

Supplementary table 2: M1

Results of unconditional model testing for association between a linear and quadratic term for mean centred age and outcome* (M1) in a sample with complete exposure, outcome and confounding data (n=14013)

* SDQ subscale scores at each timepoint, clustered within individuals

Subscales	Linear Mean difference (95%CI), p-value	Quadratic Mean difference (95%CI), p-value
Emotional symptoms	0.042 (0.39-0.045), <0.0001	-0.005 (-0.060 - -0.004), <0.0001
Peer problems	0.122 (0.120-0.126), <0.0001	-0.006 (-0.006 - -0.005), <0.0001
Conduct problems	-0.064 (-0.066 - -0.061), <0.0001	0.008 (0.008-0.009), <0.0001
Hyperactivity	-0.065 (-0.068 - -0.061), <0.0001	-0.007 (-0.007 - -0.006), <0.0001

Supplemental Figure 1

Trajectories of SDQ conduct problem subscale scores for children admitted (solid line) and not admitted (dashed line) to NNU with 95% confidence intervals. Trajectories derived from the fully adjusted model including an interaction between NNU admission and linear age (p=0.118)

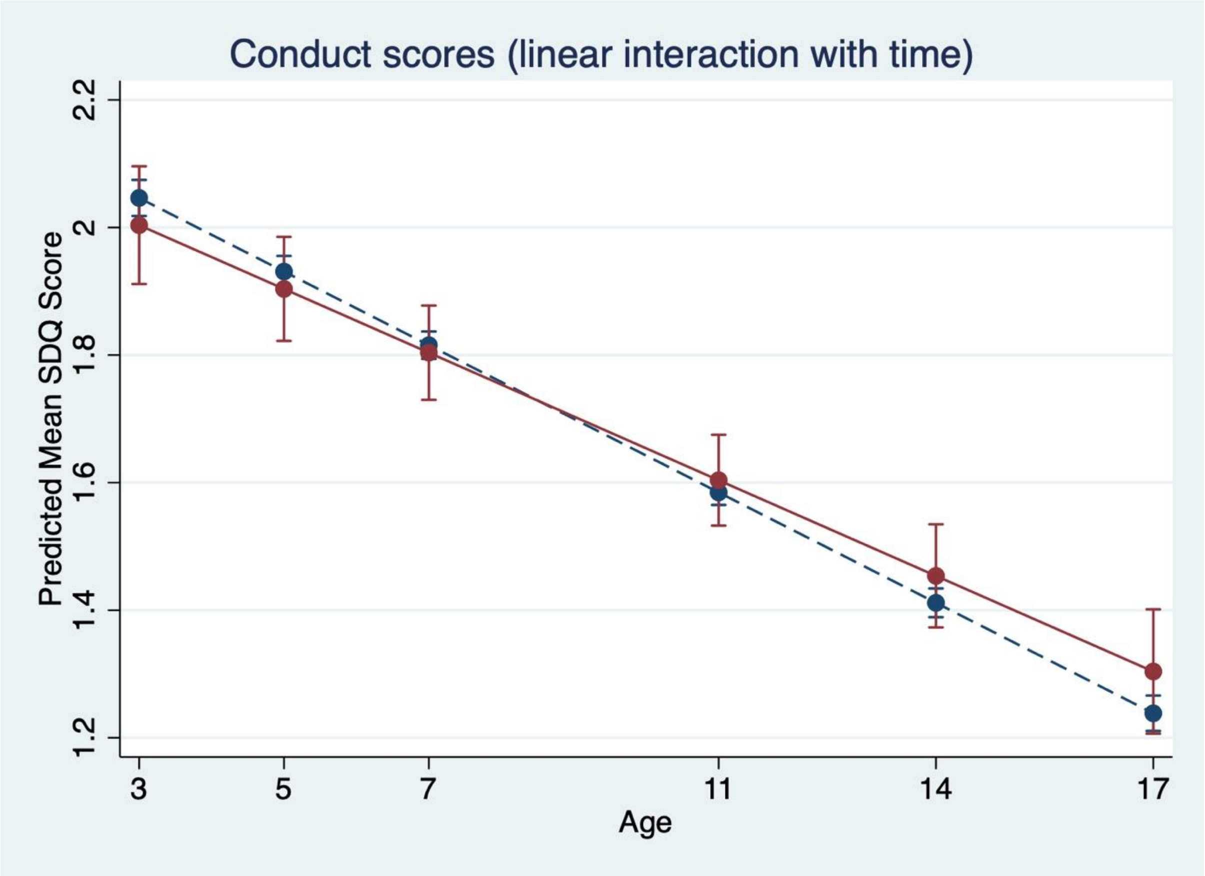

Results from sensitivity analyses based on child-reported measures in adolescence

Child-reported depressive symptoms at 14 years as measured by the sMFQ

In sensitivity analyses using the sMFQ at age 14 years as the outcome (n=14,013) there was no evidence of an association between NNU admission and higher depressive symptom scores in the unadjusted model (MD: 0·24, 95% CI: -0·22 to 0·70, p=0·309), however there was evidence of an association in the fully adjusted model (MD: 0·56, 95% CI: 0·04 to 1.08, p=0·034), with child sex resulting in unmasking of this association suggesting the presence of negative confounding.

Child-reported psychological distress at 17 years as measured by the K6

Insensitivity analyses using the K6 at age 17 years as the outcome (n=14,013), no evidence of an association was found between NNU admission and higher psychological distress scores at the age of 17 years in the unadjusted (MD: 0.25, 95% CI: -0·15 to 0·65, p=0·218) or adjusted models (MD: 0.32, 95% CI: -0·13 to 0·77, p=0·162).

Supplemental table 3: MFQ and Kessler 6

Univariable and multivariable linear regression analyses for the association between admission to neonatal unit and Short Moods and Feelings Questionnaire (MFQ) scores at age 14 years and Kessler 6 scores at age 17 years. Based on participants with complete exposure, confounders and at least one SDQ outcome available (n=14,013)

	**MFQ at 14 years Mean Difference (95% CI), p-value**	**Kessler-6 at 17 years Mean Difference (95% CI), p-value**
M1	0.24 (-0.22, 0.70), p=0.309	0.25 (-0.15, 0.65), p=0.218
M2	0.62 (0.11, 1.12), p=0.017	0.38 (-0.07, 0.83), p=0.099
M3	0.61 (0.11, 1.11), p=0.018	0.33 (-0.11, 0.78), p=0.139
M4	0.58 (0.08, 1.09), p=0.024	0.33 (-0.12, 0.78), p=0.147
M5	0.56 (0.04, 1.08), p=0.034	0.32 (-0.13, 0.77), p=0.162

M1 univariable model (unadjusted model)
M2 M2 + child related factors: gestational age, birthweight, sex and ethnicity
M3 M3 + socio-economic and maternal demographics: parental social class, weekly income, maternal age at birth and maternal education
M4 M4 + maternal prenatal factors: maternal lifetime history of depression, smoking in pregnancy, alcohol consumption in pregnancy, multiple pregnancy, whether antenatal care was given, pre-pregnancy BMI
M5 M5 + maternal perinatal factors: complications in labour and pregnancy and delivery type

Supplemental Table 4: Analyses restricted to children born after 34 weeks of gestation

Table presenting results of sensitivity analyses restricted to participants in the sample who were born at 34+ weeks of gestation (n=13,746).

Regression models:	Outcome: child mental health problems age 3 to 17 years, measured with Strength and Difficulties Questionnaire sub-scales			
	Emotional symptoms	Peer problems	Conduct problems	Hyperactivity
	Mean difference (95% CI), p-value	Mean difference (95% CI), p-value	Mean difference (95% CI), p-value	Mean difference (95% CI), p-value
Model 6: Model 5 + complications in labour and pregnancy and delivery type	0.14 (0.05 to 0.23), p=0.002	0.10 (0.02 to 0.18), 0.015	0.01 (-0.07 to 0.08), 0.930	0.04 (-0.07 to 0.15), 0.480

Supplemental Table 4: Analyses in multiply imputed dataset

Table presenting results of sensitivity analyses in models including participants with complete exposure and at least one outcome measurement, with missing confounding variables imputed (n=16,432)

Regression models:	Outcome: child mental health problems age 3 to 17 years, measured with Strength and Difficulties Questionnaire sub-scales			
	Emotional symptoms	Peer problems	Conduct problems	Hyperactivity
	Mean difference (95% CI), p-value	Mean difference (95% CI), p-value	Mean difference (95% CI), p-value	Mean difference (95% CI), p-value
Model 6: Model 5 + complications in labour and pregnancy and delivery type	0.11 (0.02 to 0.19), p=0.012	0.10 (0.02 to 0.17), 0.010	-0.02 (-0.09 to 0.05), 0.585	0.02 (-0.08 to 0.12), 0.697

Child-reported emotional and behavioural difficulties at 17 years as measured by the SDQ

In sensitivity analyses using the child reported SDQ scores at 17 years as the outcome (n=14,013), there was no evidence of an association between NNU admission and higher emotional scores in the adjusted (MD 0.02, 95% CI -0.18 to 0.23, p=0.824) or unadjusted models (MD 0.02, 95% CI -0.21 to 0.25, p=0.831). The same was true of the peer relationship scores (unadjusted MD 0.10, 95% ci -0.06 to 0.27, p=0.225; adjusted MD 0.04, 95% CI -0.15 to 0.22, p=0.694), conduct scores (unadjusted MD 0.18, 95% CI -0.03 to 0.39, p=0.089; adjusted MD 0.16, 95% CI -0.08 to 0.38, p=0.181) and hyperactivity scores (unadjusted MD 0.004, 95% CI -0.13 to 0.13, p=0.943; adjusted MD 0.02, 95% CI -0.13 to 0.17, p=0.782).

Supplementary table 4: Child-reported SDQ

Univariable and multivariable linear regression analyses for the association between admission to neonatal unit and child reported Strengths and Difficulty Questionnaire scores at 17 years. Based on participants with complete exposure, confounders and at least one SDQ outcome available (n=14,013)

	Emotional symptoms Mean Difference (95% CI), p-value	Peer problems Mean Difference (95% CI), p-value	Conduct problems Mean Difference (95% CI), p-value	Hyperactivity Mean Difference (95% CI), p-value
M1	0.02 (-0.18, 0.23), p=0.824	0.10 (-0.06, 0.27), p=0.225	0.004 (-0.13, 0.13), p=0.943	0.18 (-0.03, 0.39), p=0.089
M2	0.06 (-0.16, 0.29), p=0.566	0.05 (-0.13, 0.24), p=0.578	0.02 (-0.12, 0.17), p=0.757	0.19 (-0.04, 0.42), p=0.097
M3	0.05 (-0.18, 0.27), p=0.685	0.06 (-0.13, 0.24), p=0.547	0.02 (-0.13, 0.16), p=0.800	0.18 (-0.05, 0.41), p=0.123
M4	0.04 (-0.18, 0.27), p=0.715	0.04 (-0.14, 0.23), p=0.633	0.02 (-0.12, 0.17), p=0.767	0.17 (-0.06, 0.40), p=0.139
M5	0.02 (-0.21, 0.25), p=0.831	0.04 (-0.15, 0.22), p=0.694	0.02 (-0.13, 0.17), p=0.782	0.16 (-0.08, 0.38), p=0.181

M1 univariable model (unadjusted model)
M2 M2 + child related factors: gestational age, birthweight, sex and ethnicity
M3 M3 + socio-economic and maternal demographics: parental social class, weekly income, maternal age at birth and maternal education
M4 M4 + maternal prenatal factors: maternal lifetime history of depression, smoking in pregnancy, alcohol consumption in pregnancy, multiple pregnancy, whether antenatal care was given, pre-pregnancy BMI
M5 M5 + maternal perinatal factors: complications in labour and pregnancy and delivery type

References

(1) Angold A, Stephen C. Development of a short questionnaire for use in epidemiological studies of depression in children and adolescents. Age (years) 1995;6(11):237-249.

(2) Thapar A, McGuffin P. Validity of the shortened Mood and Feelings Questionnaire in a community sample of children and adolescents: a preliminary research note. Psychiatry Res 1998;81(2):259-268.

(3) Rhew IC, Simpson K, Tracy M, Lymp J, McCauley E, Tsuang D, et al. Criterion validity of the Short Mood and Feelings Questionnaire and one-and two-item depression screens in young adolescents. Child and adolescent psychiatry and mental health 2010;4(1):1-11.

(4) Kessler RC, Barker PR, Colpe LJ, Epstein JF, Gfroerer JC, Hiripi E, et al. Screening for serious mental illness in the general population. Arch Gen Psychiatry 2003;60(2):184-189.

(5) Mewton L, Kessler RC, Slade T, Hobbs MJ, Brownhill L, Birrell L, et al. The psychometric properties of the Kessler Psychological Distress Scale (K6) in a general population sample of adolescents. Psychol Assess 2016;28(10):1232.

(6) Green JG, Gruber MJ, Sampson NA, Zaslavsky AM, Kessler RC. Improving the K6 short scale to predict serious emotional disturbance in adolescents in the USA. International journal of methods in psychiatric research 2010;19(S1):23-35.

(7) Chan SM, Fung TCT. Reliability and validity of K10 and K6 in screening depressive symptoms in Hong Kong adolescents. Vulnerable Children and Youth Studies 2014;9(1):75-85.

(8) Ferro MA. The psychometric properties of the Kessler Psychological Distress Scale (K6) in an epidemiological sample of Canadian youth. The Canadian Journal of Psychiatry 2019;64(9):647-657.

(9) Peiper N, Clayton R, Wilson R, Illback R. The performance of the K6 Scale in a large school sample. Psychol Assess 2015;27(1):228.

(10) Plewis I, Calderwood L, Hawkes D, Hughes G, Joshi H. Millennium Cohort Study: technical report on sampling. London: Centre for Longitudinal Studies 2007.
