# Supplementary material for: Neonatal unit admission and offspring mental health trajectories across childhood and adolescence: a nationally representative UK cohort study
Source: BMJ Paediatr Open. 2025 Jan 28;9(1):e003092. doi: 10.1136/bmjpo-2024-003092 (PMC11781137; doi:10.1136/bmjpo-2024-003092)
Supplement: Supplementary file 2 [file bmjpo-2024-003092.reviewer_comments.pdf]

PEER REVIEW HISTORY

BMJ Paediatrics Open publishes all reviews undertaken for accepted manuscripts. Reviewers are asked to complete a checklist review form and are provided with free text boxes to elaborate on their assessment. These free text comments are reproduced below.

ARTICLE DETAILS

**TITLE (PROVISIONAL)**	Neonatal unit admission and offspring mental health trajectories across childhood and adolescence: a nationally representative UK cohort study
**AUTHORS**	Nandakumar, Madura; Lewis, Gemma; Lewis, Glyn; Solmi, Francesca; Srinivasan, Ramya

VERSION 1 - REVIEW

**REVIEWER NAME**	***Heather Burris***
**REVIEWER AFFILIATION**	None disclosed
**REVIEWER CONFLICT OF INTEREST**	N/A
**DATE REVIEW RETURNED**	01-Nov-2024

**GENERAL COMMENTS**	Thank you for the opportunity to review this revised submission on NNU admission and longer term outcomes.<br><br>Major: While the proportion of children with brain injury or genetic syndromes is low in the population at large, they are not rare among term infants in neonatal intensive care units. This comment remains incompletely addressed. Are there data on re-hospitalization? These children are often readmitted. In other words,<br>is NNU admission in the absence of subsequent hospitalization still a risk factor for poor outcomes?<br>Minor:<br>In abstract, no need to go to 2 decimal places for %. 48.9% is fine, for example. Also recommend changing throughout manuscript.<br>Gestational age can be rounded to nearest day (no need for decimals)<br><br>Suggest "infants" instead of "babies" throughout (less colloquial).<br><br>I don't think the unconditional model is particularly helpful. Suggest instead starting with model 1 (independent variable == NNU, dependent variable outcome variables, adjusted for age at visit and age^2)<br>Then can eliminate Supplemental Table 2. It is not interesting to know that age and age squared are associated with the outcome. Perhaps prior literature has shown this and can be cited and the justified to be included a priori.<br><br>Adjusting for both birthweight and gestational age is problematic (collinearity). If planning to do that, suggest using gestational age and then BWT for GA Z score.

**REVIEWER NAME**	***Marlous Hall***
**REVIEWER AFFILIATION**	None disclosed
**REVIEWER CONFLICT OF INTEREST**	Not applicable
**DATE REVIEW RETURNED**	14-Nov-2024

**GENERAL COMMENTS**	All of the statistical analyses aspects raised in my previous review have been addressed and incorporated into the updated manuscript, and I have no further recommendations to make. The authors present a well conducted analyses of these data, which is clearly and thoroughly reported according to the STROBE reporting standards, analyses are appropriate with respect to the aims of the study and suitable sensitivity analyses have been conducted and appropriately interpreted.

VERSION 1 – AUTHOR RESPONSE

Reviewer: 1, Heather Burris

Comments to the Author

Thank you for the opportunity to review this revised submission on NNU admission and longer term outcomes.

Major:

While the proportion of children with brain injury or genetic syndromes is low in the population at large, they are not rare among term infants in neonatal intensive care units. This comment remains incompletely addressed. Are there data on re-hospitalization? These children are often readmitted. In other words,is NNU admission in the absence of subsequent hospitalization still a risk factor for poor outcomes?

We agree with the reviewer that the proportion of children with brain injury or genetic syndromes will be higher in the neonatal units in question and that this is a limitation of this study. The reviewer proposes an interesting method to account for this within the current study, but this data is unfortunately not available within the sample. However, in order to highlight this point we have amended the discussion to emphasise this on page 12 lines 300-309.

“Similarly, we were unable to directly control for the reason the child required NNU admission, which could include brain injuries or genetic conditions, particularly in children born at term and are also likely to be higher in those admitted to NNU. Whilst we have included a number of variables which might be associated with these outcomes (e.g. maternal age, antenatal visit attendances, pregnancy and birth complications) we cannot exclude presence of brain injuries or genetic conditions as a potential confounder of these associations. Furthermore, if these infants are more likely to experience repeated hospitalisations and if those were associated with the outcomes under investigation as opposed to NNU in itself our results might be affected by additional residual confounding.”

Minor:

In abstract, no need to go to 2 decimal places for %. 48.9% is fine, for example. Also recommend changing throughout manuscript. Gestational age can be rounded to nearest day (no need for decimals)

We have amended this accordingly

Suggest "infants" instead of "babies" throughout (less colloquial).

We have amended this accordingly.

I don't think the unconditional model is particularly helpful. Suggest instead starting with model 1 (independent variable == NNU, dependent variable outcome variables, adjusted for age at visit and age^2). Then can eliminate Supplemental Table 2. It is not interesting to know that age and age squared are associated with the outcome. Perhaps prior literature has shown this and can be cited and the justified to be included a priori.

We agree that, on their own, unconditional models do not help answer the main research question under investigation. For this reason, we currently include them in supplemental material. However, we would prefer to retain those for transparency as they are needed to understand how to best model time in the context of our analyses and, as such, we believe constitute an important part of our modelling approach.

Adjusting for both birthweight and gestational age is problematic (collinearity). If planning to do that, suggest using gestational age and then BWT for GA Z score.

The correlation between birthweight and gestational age in the sample is 0.58 i.e. a moderate correlation. We have included birthweight and gestational age in the models only as confounders, so we are not interested in their individual coefficients and are only concerned with effective reduction of confounding. Moderate collinearity between two confounding variables does not typically interfere with the estimation of the primary association of interest i.e. that between exposure and outcome. This means that it is possible to include both birthweight and gestational age in the models with minimal impact on the primary association of interest. We have included the correlation between these two variables in the relevant section of the supplement (page 4).

“Birthweight and gestational age in the sample were moderately correlated (0.59). As a result, given these variables are being considered only as confounders, to effectively reduce confounding, we included both variables in the models. This is because moderate collinearity between two confounding variables does not typically interfere with the estimation of the primary association of interest, between the exposure and outcome.”

Reviewer: 2, Marlous Hall

Comments to the Author

All of the statistical analyses aspects raised in my previous review have been addressed and incorporated into the updated manuscript, and I have no further recommendations to make. The authors present a well conducted analyses of these data, which is clearly and thoroughly reported according to the STROBE reporting standards, analyses are appropriate with respect to the aims of the study and suitable sensitivity analyses have been conducted and appropriately interpreted.

VERSION 2 – REVIEW

**REVIEWER NAME**	***Marlous Hall***
**REVIEWER AFFILIATION**	None disclosed
**REVIEWER CONFLICT OF INTEREST**	Not applicable
**DATE REVIEW RETURNED**	05-Dec-2024

**GENERAL COMMENTS**	All comments have been suitably addressed and I have no further recommendations to make.
